# Medical Complicity in Torture at Guantánamo Bay: Evidence Is the First Step Toward Justice

**DOI:** 10.1371/journal.pmed.1001028

**Published:** 2011-04-26

**Authors:** The PLoS Medicine Editors

## Abstract

The *PLoS Medicine* editors argue that the publication of new evidence of the complicity of medical personnel in torture at Guantánamo Bay (GTMO) should bolster calls for further investigation into the role of doctors at the prison.

This month *PLoS Medicine* publishes a research article reporting that medical doctors and mental health professionals from the US Department of Defense working at the Guantánamo Bay prison concealed or failed to document medical evidence of intentional harm of nine detainees [1].

The authors—Vincent Iacopino, a senior medical advisor to Physicians for Human Rights, and Stephen Xenakis, a retired US Army Brigadier General—present a case series of nine individuals detained in Guantánamo Bay, all of whom alleged torture and ill treatment during detention at the facility. Acting as nongovernmental medical personnel recruited by lawyers acting for the nine detainees, the authors scrutinized medical records, client affidavits, attorney–client notes and summaries, and legal declarations of medical experts for evidence of torture and ill treatment. Where such evidence existed, Iacopino and Xenakis assessed whether medical personnel at the camp had either documented or treated symptoms arising from torture.

Guantánamo Bay is a detention facility for US prisoners in Cuba that opened in 2002. Despite US President Barack Obama pledging during the 2008 presidential elections to shut Guantánamo Bay and transfer detainees to the US for trial, the prison shows no sign of closing [2] and 172 detainees remain incarcerated there. Both authors of the paper, which publishes this week, are from Physicians for Human Rights, a US not-for-profit organization dedicated to mobilizing health professionals to investigate and stop human rights abuses, that has previously documented in a series of extensive reports evidence of the involvement of US personnel in torture at Guantánamo Bay and other prisons worldwide [3],[4],[5].

This research article adds solid, specific evidence of both human rights abuses at Guantánamo Bay and the apparent complicity of medical personnel in the abuse. It documents specific features of torture alleged by the detainees including “enhanced interrogation techniques” that were deemed suitable for use in US interrogations by then-President George W. Bush [6]. These techniques included sleep deprivation, exposure to temperature extremes, serious threats, forced positions, beatings, and forced nudity. In addition, each of the nine detainees reported being subjected to severe beatings, sexual assault and/or the threat of rape, mock execution, mock disappearance, and being choked. By analyzing medical records and carrying out psychological evaluations, either directly or by proxy, the authors of the *PLoS Medicine* paper were able to detail the physical and mental health of each detainee. Crucially, although some of the physical injuries sustained by detainees that were consistent with allegations of torture were documented by medical personnel in the camp, *causes* of injury were not investigated by those personnel. Furthermore, mental health practitioners in the camp recorded symptoms characteristic of post-traumatic stress disorder (PTSD) in seven of nine detainees, but failed to investigate the causes of the symptoms or to diagnose or treat the detainees' PTSD.

Iacopino and Xenakis reach a sobering conclusion: “Medical doctors and mental health personnel assigned to the US Department of Defense neglected and/or concealed medical evidence of intentional harm. The full extent of medical complicity in US torture practices will not be known until there is a thorough, impartial investigation including relevant classified information. We believe that, until such time as such an investigation is undertaken, and those responsible for torture are held accountable, the ethical integrity of medical and other healing professions remains compromised.”

These findings are of course at stark odds with the codes of international medical associations, which prohibit physicians from participating in or being present during torture or other degrading procedures [7],[8]. Their findings especially violate the American Medical Association's stance that specifically charges physicians with providing assistance to victims of torture, and to “whenever possible, strive to change situations in which torture is practiced or the potential for torture is great” [8].

Publishing peer-reviewed documentary evidence of harm—especially from settings difficult to access such as prisons or conflict settings—is a vital and important role of medical journals. This paper adds new evidence that will bolster calls for further investigation into the complicity of medical personnel in torture at Guantánamo Bay, which clearly breaches fundamental human rights. Evidence must be obtained and properly reported in order that those who have been negligent (or worse) can be appropriately dealt with, including where necessary by prosecution.

## References

[1] Iacopino V, Xenakis SN (2011). Neglect of medical evidence of torture in Guantánamo Bay:
A case series.. PLoS Med.

[2] [No authors listed] (7 March 2011). Barack Obama restarts Guantánamo trials.
Guardian.co.uk.. http://www.guardian.co.uk/world/2011/mar/07/guantanamo-bay-trials-restart.

[3] Physicians for Human Rights (2005). Break them down: Systematic use of psychological torture by US
forces.. http://physiciansforhumanrights.org/library/documents/reports/break-them-down-the.pdf.

[4] Physicians for Human Rights (2007). Leave no marks: Enhanced interrogation techniques and the risk of
criminality.. http://physiciansforhumanrights.org/library/report-2007-08-02.html.

[5] Physicians for Human Rights (2008). Broken laws, broken promises.. http://brokenlives.info/?page_id=69.

[6] J. Bybee, Assistant Attorney General, Office of Legal Counsel, Department of Justice (1 August 2002) Memorandum for A. Gonzales. Counsel to the President. Available: http://www.washingtonpost.com/wp-srv/nation/documents/dojinterrogationmemo20020801.pdf. Accessed 4 March 2011

[7] World Medical Association (2009). World Medical Association Council Resolution on Prohibition of
Physician Participation in Torture.. http://www.wma.net/en/30publications/10policies/30council/cr_8/index.html.pdf?print-media-type&footer-right=[page]/[toPage].

[8] American Medical Association (1999). Code of medical ethics: Opinion
2.067—Torture.. http://www.ama-assn.org/ama/pub/physician-resources/medical-ethics/code-medical-ethics/opinion2067.shtml.

